# Developing microRNA screening as a functional genomics tool for disease research

**DOI:** 10.3389/fphys.2013.00223

**Published:** 2013-08-27

**Authors:** Derek Lemons, Mano R. Maurya, Shankar Subramaniam, Mark Mercola

**Affiliations:** ^1^Department of Bioengineering, Jacobs School of Engineering, University of CaliforniaSan Diego, La Jolla, CA, USA; ^2^Muscle Development and Regeneration Program, Sanford-Burnham Medical Research InstituteLa Jolla, CA, USA

**Keywords:** systems biology and network biology, microRNA target, protein-protein interaction, functional genomics, functional screens, proteomics

## Abstract

Originally discovered as regulators of developmental timing in *C. elegans*, microRNAs (miRNAs) have emerged as modulators of nearly every cellular process, from normal development to pathogenesis. With the advent of whole genome libraries of miRNA mimics suitable for high throughput screening, it is possible to comprehensively evaluate the function of each member of the miRNAome in cell-based assays. Since the relatively few microRNAs in the genome are thought to directly regulate a large portion of the proteome, miRNAome screening, coupled with the identification of the regulated proteins, might be a powerful new approach to gaining insight into complex biological processes.

## Introduction

Transcriptomics, proteomics and other ‘omics data describing biological phenomena are amassing at an astounding rate that was unimaginable even a few years ago. In principle, researchers will be able to utilize these data to formulate and answer complex biological questions—including important questions in cardiovascular medicine. The amount of primary data is growing exponentially with the availability of disease-specific assays and powerful new technologies, such as Next-Gen Sequencing (NGS aka RNA-Seq) (Marioni et al., 2008; Wang et al., 2009), ChiP-SEQ (Johnson et al., 2007), protein microarrays (Melton, 2004; Mattoon and Schweitzer, 2009), and mass-spectroscopy-based proteomics (Hernandez et al., 2006). As of November 2012, the Gene Expression Omnibus (http://www.ncbi.nlm.nih.gov/geo/) lists 2720 datasets covering over 800,000 assays while ArrayExpress at European Bioinformatics Institute contains data from 33,868 datasets covering nearly a million assays (http://www.ebi.ac.uk/arrayexpress/). Moreover, advances in computational algorithms to identify putative connections among nodes have magnified the effect, making the sum total of ‘omics information seemingly intractable. For example, the Human Protein Reference Database (http://www.hprd.org) (Keshava Prasad et al., 2009) contains information on a daunting 41,327 protein-protein interactions (PPIs), and this is probably a lower estimate. Making sense of the primary and derived information is arguably one of the largest challenges in systems biology.

One approach is to use high throughput biological screening technology to probe the nodes and networks, providing experimental validation of the computationally determined networks. Nearly five decades ago, the pharmaceutical industry refocused its efforts on screening and has since developed advanced technology, expertise, and chemical libraries, accelerating the production of new drugs that have had an enormous impact on longevity and quality of life (Kaye and Krum, 2007). A recent byproduct of this activity has been the adoption of high throughput screening approaches in academia. Although the original screening applications were target-centric, essentially designed to discover molecules that interact with a known target, the last decade has seen the development of assays designed to explore complex biological mechanisms including assays based on human induced pluripotent stem cells (hiPSCs) to model cardiovascular disease (Nsair and MacLellan, 2011; Mercola et al., 2013). Such assays are typically phenotypic, meaning that they read out morphology, behavior or physiology of cells in culture or even in whole organisms such as zebrafish or Drosophila. The advantage of phenotypic screening as a discovery tool is that it probes a plethora of biomolecules involved in a given phenotype. Phenotypic screening coupled to the identification of cellular proteins or genes targeted in the screens is termed “chemical” or “functional” genomics, depending on whether the library is a chemical or a nucleic acid, respectively, by analogy to the unbiased evaluation of the genome by classical “forward” genetic screening by mutagenesis (Stockwell, 2000).

In this review, we discuss functional genomics technologies for identifying cellular proteins and genes of interest, and application of these approaches to sift through and validate the vastness of information to gain meaningful insight into mechanisms of complex phenotypes and diseases. Key among the technologies is RNA interference (siRNA or shRNA) technology, which has proven to be a powerful method to evaluate the function of candidate genes, and even screen entire genomes to reveal pathway components that govern complex processes, including stem cell identity (Chia et al., 2010) and sensitization of tumor cells to chemotherapeutics (Whitehurst et al., 2007). By probing all genes, whole-genome RNAi strategies offers a comprehensive alternative to chemical screening to interrogate the vastness of the proteome, estimated at over 1,000,000 total human proteins, including splice variants, post-translational modifications and somatic mutations (Jensen, 2004). This number greatly overshadows the calculated 3000–10,0000 so-called “druggable” proteins, that have topologically defined drug-binding pockets that are considered desirable, which includes enzymes, GPCRs, kinases, nuclear receptors and ion channels (Overington et al., 2006). Targeting only these classes, however, ignores many biologically interesting proteins that play important roles in disease, such as transcription factors and scaffold proteins (Stockwell, 2000; Crews, 2010).

**Figure 1:**
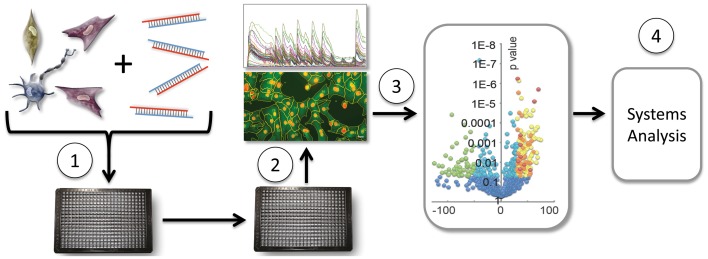
**Moderate throughput screening of miRNAs in cell-based assays.** Cells are transfected with individual miRNAs from a miRNAome library in 384-well or other multiwell format (1). Following culture, either image-based (shown) or plate-reader acquisition of data, and subsequent analysis (2), profiles miRNAs by activity shown in a volcano plot (3), providing a dataset for network analysis (4) and Figure 2.

In addition to unbiased siRNA or shRNA screens, we explore the concept that miRNA screening might be a particularly promising means of identifying critical proteins in biological control networks. miRNAs are endogenous, ~22-nucleotide single-stranded RNAs that selectively bind and suppress multiple mRNA targets in the context of the RNA-Induced Silencing Complex (miRISC). There are only about 2000 known miRNAs in the human genome (http://www.mirbase.org), yet they are estimated to regulate 60% of the total proteome (Friedman et al., 2009). By governing translation and mRNA stability, miRNAs fine-tune nearly every normal and pathological process examined (Filipowicz et al., 2008; Bartel, 2009). In cardiovascular biology, miRNAs control early embryonic development and adult disease, exemplified by the essential roles of miR-1 and miR-133 in heart development (Zhao et al., 2007; Liu et al., 2008) and miR-21 and miR-208a in cardiac remodeling after myocardial infarction (Van Rooij et al., 2007; Thum et al., 2008) and metabolism (Grueter et al., 2012). Given their evolutionarily conserved, and arguably optimized, role in regulating proteins that occupy critical nodes in networks controlling complex biology (Shreenivasaiah et al., 2010), we postulate that screening with miRNA libraries could be used to elucidate disease-modifying mechanisms (Figure 1). At least conceptually, the outcome of a miRNA screen can be informative regardless of whether or not a particular miRNA is normally involved in the process being probed. On the one hand these screens may identify miRNAs that normally modulate biological phenomena, adding new dimensions to the miRNAome. On the other hand, miRNAs, when ectopically expressed, will downregulate proteins they do not normally regulate in a native biological context. Thus, miRNA screening, like chemical library screening, can reveal key regulatory proteins that elicit a given phenotype. One major roadblock is the limited ability to identify high confidence targets of miRNAs. If emerging technologies can overcome this issue, miRNA screening might become a tremendously powerful approach to elucidating systems-level control networks and identifying critical node proteins that might be ideally poised as drug targets. In this review we discuss the current technologies for functional miRNA screening and target identification, and consider the challenges that must be resolved in order to achieve the potential offered by the approach.

## Functional genomics technology

Oligonucleotide libraries offer an alternative to chemical libraries for probing cardiovascular or other disease phenotypes. RNA interference (siRNA or shRNA) technology functions by introducing a double stranded small interfering (siRNA) or short hairpin (shRNA) RNA into the cell that basepairs with cognate mRNAs in the RNA-induced Silencing Complex (RISC), targeting the mRNAs for degradation.

Advances in oligonucleotide chemistry have improved siRNA technologies. For instance, modifying the second position of siRNAs with 2'-O-methyl linkage significantly reduces off-target effects that result when siRNAs act like miRNAs (i.e. target imprecisely base-paired mRNAs for downregulation by the RISC) (Jackson et al., 2006). Other chemical or sequence modifications made to the ends of the oligonucleotide strands dictate which strand of the oligonucleotide duplex become packaged into RISC, reducing off-target effects caused by the complementary strand (Schwarz et al., 2003). Furthermore, it has become common to screen pools of multiple siRNAs against a single mRNA target to increase the likelihood of eliciting a phenotypic effect (Parsons et al., 2009). Modern commercial siRNA libraries use these technologies to provide specific and potent knockdown of target genes. Examples of genome-wide siRNA screening libraries include Stealth RNAi™ and Silencer Select (Life Technologies), ON-TARGETplus and siGENOME (ThermoScientific), AccuTarget (Bioneer), and MISSION® siRNA (Sigma-Aldrich).

Compared to standard siRNAs, short hairpin RNA (shRNA) offers multiple advantages. This technology uses lessons learned from miRNA research, harnessing the cell's miRNA biogenesis machinery to process the hairpin into specific siRNA duplexes. And, unlike many miRNAs, the shRNA sequences are typically optimized to ensure only one strand becomes packaged into RISC. shRNA is most commonly delivered to cells by transfection or infection using plasmid or viral vectors capable of providing long-lasting downregulation of target genes. The first shRNA libraries used RNA Polymerase III to transcribe the hairpin sequence (Berns et al., 2004; Moffat et al., 2006). Subsequent studies, however, showed that design based on primary miRNA transcripts (pri-miRNA) gave improved efficiency of siRNA packaging into RISC (Chang et al., 2006). Additionally, primary miRNA transcript-based shRNAs are expressed via RNA Polymerase II, allowing co-expression of fluorescent or drug-selectable transgene markers from a single promoter. Another powerful advance in shRNA technology is the use of pooled barcoded shRNAs combined with high throughput sequencing deconvolution, circumventing the need for multi-well plates, liquid handling robots, and large amounts of reagents (Sims et al., 2011). A variety of libraries are available commercially, each utilizing slightly different design strategies and delivery vectors. Examples include MISSION® (Sigma-Aldrich), BLOCK-iT™ (Life Technologies) DECIPHER (Cellecta – Free to academia), and Decode Pooled Lentiviral shRNAs (Thermo Scientific).

## Logic of miRNAs as screening tools

miRNAs make an intriguing starting point for phenotypic screening, as they have many desirable qualities that may allow identification of pathways or networks involved in a particular process that might not be found using single gene screening methods. miRNAs co-evolved to regulate expression of the transcriptome and proteome, and therefore have selective relationships with their targets and the processes they regulate. Indeed, it is thought that entire genomes have adjusted to the pool of miRNAs in each organism by selectively removing potential target sites that, if present in transcripts, would cause undesirable downregulation that would be detrimental to the organism (Stark et al., 2005). Perhaps the most useful aspect of miRNA-genome co-evolution is that each miRNA typically targets numerous genes. Varying estimates have been suggested using computational target predictions as guidelines, but most telling is that expression profiles after miRNA overexpression or removal indicates that a large portion of the transcriptome/proteome is under the control of miRNAs, with each miRNA potentially regulating on the order of hundreds of proteins (Filipowicz et al., 2008; Selbach et al., 2008; Bartel, 2009; Friedman et al., 2009; Shirdel et al., 2011). For instance, miR-223 is estimated by proteomics to affect the expression of more than 200 genes in neutrophils alone (Baek et al., 2008). On the other hand, deletion of certain miRNAs cause no discernible developmental phenotypes (Miska et al., 2007; Alvarez-Saavedra and Horvitz, 2010), indicating that they affect only a small number of targets which are relatively specialized or that their effect on their targets is only a small percentage of the total expression level. These miRNAs, especially those that are evolutionary ‘newborns’ (i.e. found only in one species or genus), may function mainly to buffer expression of their targets against fluctuation due to intrinsic and extrinsic factors, and have for this reason been termed “canalizing” miRNAs (Wu et al., 2009).

From a systems biology and drug target identification perspective, the most remarkable feature of miRNAs is that they often target proteins at the nodes of important regulatory pathways (Shreenivasaiah et al., 2010; Ichimura et al., 2011). Moreover, many miRNAs, especially those conserved within vertebrates, govern multiple proteins within a single pathway (Cui et al., 2006; Ichimura et al., 2011; Sass et al., 2011; Shirdel et al., 2011). Consequently, these miRNAs function as physiological or developmental switches that fine-tune the proteome of a given cell or tissue. Specific cases include the regulation of Wnt signaling components by miR-34 (Kim et al., 2011), regulation of alternative splicing by miR-23 (Kalsotra et al., 2010), regulation of the p53 network by miR-125b (Le et al., 2011), regulation of phosphatidylinositol- 3-OH kinase (PI(3)K)–AKT signaling (Small et al., 2010), and suppression of smooth muscle specific proteins in cardiomyocytes (Liu et al., 2008). miR-21 targets PPAR alpha pathway in modulating flow-induced endothelial inflammation (Zhou et al., 2011) and miR-23b is involved in endothelial cell growth (Wang et al., 2010).

Since miRNAs govern such large-scale changes in translation, it is perhaps not surprising that they have been found to be involved in nearly every normal and pathological process examined so far (Filipowicz et al., 2008; Bartel, 2009). Given the evolutionarily strategic position of miRNAs and their ability to directly control expression of a large portion of the proteome through simultaneous targeting of multiple genes, they potentially offer an efficient means to interrogate critical processes and the potential to identify genes of interest for phenotypes which may not be affected by the single gene mutation or knockdown approaches typical of most classical genetic or even chemical biology and si/shRNA screening methods. As an example, recent whole genome miRNA screens have led to the discovery of miRNAs and target genes that allocate mesoderm and ectoderm as distinct from endoderm in the early embryo (Colas et al., 2012), modulate cardiomyocyte hypertrophy (Jentzsch et al., 2012), and regulate cell cycle re-entry of adult cardiomyocytes (Eulalio et al., 2012).

Cancer is another area where microRNA screening might reveal unanticipated therapeutic targets. For instance, recent whole-genome miRNA screen identified miR-16, miR-96, miR-182, and miR-497 as potent inhibitors of melanoma cell proliferation and viability (Poell et al., 2012), suggesting that mimics of these miRNAs optimized for use in human patients could be important therapeutic molecules. In addition to understanding the transformed state, an important aspect of cancer research where miRNA screening could be useful might be in deciphering the cellular pathways and proteins that mediate drug resistance, which could suggest combinatorial drug action, such as been recently addressed through proteomics (Erler and Linding, 2012). We expect that, in the near future, miRNA screens will discover many phenotype-modifying genes that would not and have not been identified through siRNA and chemical screens, as well as identify numerous miRNAs whose involvement in disease phenotype, progression or drug-responsiveness will provide new therapeutic targets.

**Figure 2:**
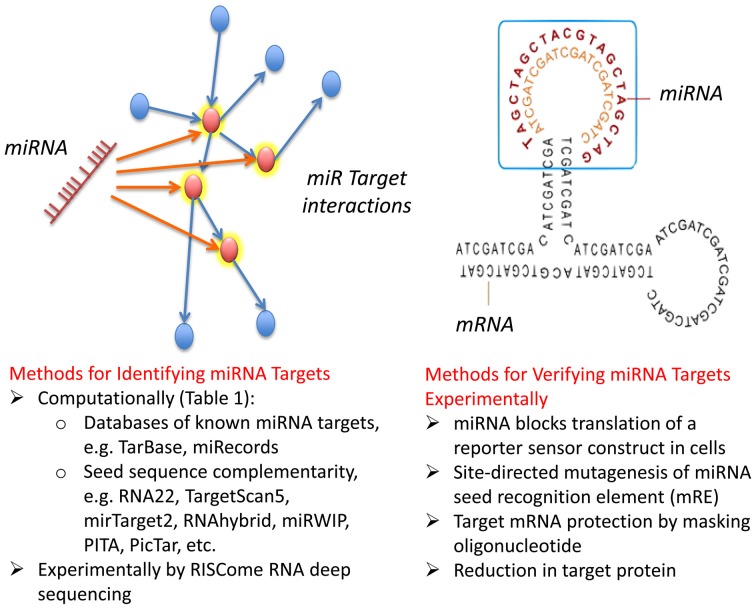
**Computational and experimental strategies to identify miRNA targets.** miRNAs target multiple proteins, and in certain instances a single family of miRNAs target multiple proteins involved in a common biological process, through imprecise basepairing with recognition sequences in mRNA (see text). Commonly used computational and biochemical approaches to identify targets are summarized along with focused strategies for confirming direct interaction of a miRNA with particular mRNA targets.

Many libraries are available commercially that allow screening using miRNA mimics either in hairpin or duplex format for the majority of known miRNAs of variety of model organisms. The oligonucleotide mimics are typically chemically modified in a manner similar to the siRNA products described above so that one strand is preferentially packaged into the RISC. Examples include Ambion® Pre-miR Precursors and miRvana™ miRNA mimics (Life Technologies), MISSION® (Sigma-Aldrich), miRIDIAN (Thermo Scientific). Unlike siRNA/shRNA screening, in which the gene affecting the phenotype is known *a priori* (although the mRNA target must be confirmed) the degeneracy of miRNA:mRNA interactions means that screening campaigns must include steps to identify the mRNA target(s) responsible for the phenotype. Below we discuss computational and biochemical methods currently used for target identification, their efficacy, and possible ways to improve the pipeline from screen dataset to target knowledge (Figure 2).

## Computational approaches to target identification

The development of computational tools for miRNA target prediction began in the early 2000's shortly after the discovery that miRNAs are pervasive members of animal genomes (Lagos-Quintana et al., 2001). Currently, many different tools are available, most utilizing a common set of concepts to inform their prediction algorithms, such as seed-match (complementarity between the 5′ of the miRNA—typically bases 2–8—and the bases in 3′ untranslated region (3′UTR) of an mRNA), evolutionary conservation of target sites and thermodynamic (free-energy) considerations for the interaction [Table1; for in depth reviews see (Alexiou et al., 2009; Xia et al., 2009; Witkos et al., 2011)].

**Table 1:** **Commonly used computational tools and algorithms for identification of miRNA targets**.

**Software/tools**	**Evolutionary conservation**	**Base-pairing/seed-match criteria**	**Surrounding sequence**	**Energy consideration**	**Additional filters/rules/learning using microarray data**
TargetScan	Across vertebrates: human, mouse and rat	7-nt (W-C complementarity for bases 2-8 of miRNA)	Seed-match extended on both sides	Yes, *z*-score to energy of miR-target interaction	No
TargetScanS	Similar; dog and chicken as well	6-nt and A-anchor	Yes	Yes	Latest version can use context information.
		G-W wobble pair allowed			
miRanda	*D. melanogaster*, *D. pseudoobscura* and *A. gambiae*; now extended to mouse, human and fish	7-nt and weighted seed-match		Yes	No
Diana-microT		5- to 7-nt, conditional	Uses a 38-nt sliding window	Yes, uses as a filter to find miRNA3′-UTR pairs	Specialized for target mRNAs with single miRNA recognition element
		G-W wobble pair and bulge allowed			
PicTar	vertebrates, flies and nematodes	7-nt		Yes	Finds common targets of several miRNAs using combinations of transcription factor binding sites.
miRTarget, miRTarget2 and miRDB	Yes	7-nt		Yes, duplex stability	Uses microarray data for positive and negative targets. SVM is used in miRTarget2 to incorporate features such as other seed types, base composition, and secondary structure.
SVMicrO	Yes	5-nt to increase sensitivity	Yes	Yes	Similar to miRTaget2. Bayesian approach is also used.

Abbreviations *W-C* *Watson-Crick* *SVM* *support-vector machine*.

The initial algorithms turned out to provide high sensitivity but low specificity (high rate of false-positives). One approach to solve this problem has been to prioritize targets predicted by multiple algorithms; however, taking the intersection (rather than union) leads to a corresponding loss of sensitivity (Alexiou et al., 2009). Developing advanced algorithms to take contextual cues into account would be a major advance. Some new algorithms strive to incorporate more comprehensive feature sets from experimental data and/or machine learning to try to improve the ratio of sensitivity to specificity. An improved version of TargetScan (Lewis et al., 2005), called TargetScanS, uses 6 instead of 7 nucleotide seed match followed by an A-anchor and incorporates information on the surrounding mRNA sequence to compute a context score which models the relative contributions of previously identified targeting features, including site type, site number, site location, local A+U content and 3′-supplementary pairing (Grimson et al., 2007; Garcia et al., 2011). An improved context-score called context+ score also considers target-site abundance and seed-pairing stability (Garcia et al., 2011). A multiple linear regression model was trained using 11 microarray data sets, and the context+ scores performed better than previous models. miRTarget2 is an improvement of the original miRTarget algorithm and uses a support-vector machine learning (SVM) algorithm to build prediction models based on a set of 131 features including seed conservation, other seed types, base composition, and secondary structure (Wang and El Naqa, 2008). SVMicrO is an SVM-based recent algorithm for miRNA target prediction in animals which tries to improve both sensitivity and specificity of prediction by using positive and negative target data for training the classifier (Liu et al., 2010). The algorithm increases sensitivity by only requiring a 5 basepair seed-match, and is trained using about 1000 positive miRNA-target pairs and microarray data-based 3500 negative miRNA-target pairs. The authors have shown a better true positive rate for SVMicrO as compared to many other popular algorithms on both the training data as well as a separate proteomic test data.

## Biochemical and proteomic approaches to target identification

Despite these advances, computational prediction of miRNA target sites in mammals are generally considered too error-prone to be used as the sole means of target identification, reviewed in Alexiou et al. (2009). We ascribe the problem to the fact that miRNA-mRNA pairing “rules” of most computational prediction algorithms were determined based on a small number of known targets discovered through genetic mutations and by observing changes in target regulation after abrogation of the interaction by site-directed mutation of the recognition sequence. As discussed above, contextual cues that influence site accessibility include sequences surrounding the recognition site and RNA-binding cofactors present in the cell. It is too soon to tell whether the innovations in algorithm design described in the preceding section will remedy this situation, but given that they are unlikely to model the influences of the cellular context, we expect that the problem of false positives and negatives will remain a serious issue. Thus, while many true targets have been discovered using various target prediction algorithms, they probably comprise a small percentage of the total regulatory network of the miRNA pathway.

## Transcriptomics and proteomics techniques

The first attempt at biochemically boot-strapping the identification of miRNA targets at a transcriptome scale assayed the total change in mRNA expression profile by microarray analysis caused by transfection of single miRNAs into human cells (Lim et al., 2005). In this case, transfection of either miR-1 or miR-124 shifted mRNA expression such that there was a greater resemblance to the natural profile of seen muscle or brain, the organs that normally express these miRNAs during development. Subsequent microarray studies looked at global changes in mRNA expression resulting from single miRNA overexpression, depletion, genetic mutants, and depletion of all miRNAs through mutations in the miRNA biogenesis pathway (Giraldez et al., 2006; Linsley et al., 2007). These early analyses proved that microarray profiling can provide a first approximation of the genes regulated by single or multiple miRNAs, consistent with the observation that the majority of changes in protein levels induced by miRNA regulation are attributable to changes in mRNA expression (Guo et al., 2010). However, as with microarray transcriptome analysis of transcription factor mutants, these analyses alone cannot reveal whether genes are the direct targets of the miRNAs, or are affected indirectly by factors downstream of the primary effector molecules. Although upregulated genes are unlikely to be directly affected by miRNA activity and can be excluded as direct targets, downregulated genes must be analyzed in greater detail to determine whether or not they are targeted directly by the miRNA(s) in question.

The simple comparison of downregulated transcript sets with the computationally predicted mRNA target sets has yielded poor correlations (Alexiou et al., 2009). While sequences of downregulated mRNAs are often enriched for “seed” complementary sequences, this is not always observed. For instance, downregulated genes lacking “seed” matches may be secondarily affected by changes in direct target genes, but they can also be direct targets which harbor less common types of miRNA target sites, such as 3′ compensatory (Brennecke et al., 2005) centered sites (Shin et al., 2010), or other non-canonical binding structures (Helwak et al., 2013). Whether a transcript is a direct target of a particular miRNA may or may not be relevant to the goals of an individual screen experiment. However, if this knowledge is required, subsequent experiments will be needed to confirm a direct miRNA:mRNA interaction. Typically, confirmation is based on abolishing regulation by mutation of the miRNA recognition site within the mRNA, and an alternative is to mask the binding site with a complementary oligoribonucleotide, preventing miRNA binding and mRNA degradation (for example, see Colas et al., 2012).

Quantitative proteomics is an analogous target discovery strategy that has gained traction in recent years, as it provides a direct readout of the ultimate effect of miRNA activity (Vinther et al., 2006; Baek et al., 2008; Yang et al., 2009, 2010; Chen et al., 2011; Yan et al., 2011). This method provides an advantage over microarray analysis, since it can detect changes in expression levels of a protein even when its cognate mRNA is not downregulated at an appreciable level. Early instances include an analysis of miR-1 in HeLa cells (Vinther et al., 2006), an analysis of miR-1, 124, and 181 in HeLa cells and miR-223 in mouse knockout neutrophils (Baek et al., 2008), and subsequent studies have examined miR-21 and miR-143 (Yang et al., 2009, 2010). An example of an advanced proteomics analysis is a recent study that used Stable Isotope Labeling by Amino acids in Cell culture (SILAC) to detect differences in protein expression induced by the overexpression of miR-34a and miR-29 (Bargaje et al., 2012). Although a number of proteins related to the biological function of the miRNAs in apoptosis were found to change, the study discusses several limitations. Chief among these is that miRNAs often only reduce target protein levels by 30–60% (Hendrickson et al., 2009) meaning that commonly applied thresholds (e.g., 2-fold) are inappropriate and a more robust statistical analysis is needed. In addition, variation in protein stability might require analyses at multiple timepoints. Finally, only about 10% of the proteins detected as downregulated by Bargaje et al. for miR-34a and miR-29 were also predicted by the consensus of 5 computational algorithms (Bargaje et al., 2012), highlighting the need for evaluating potential indirect effects (in addition to validating potential targets). Finally, as for microarray analyses, many interesting targets might be missed due to low abundance. Nevertheless, even at current depths, the recent studies suggest that proteomics analysis can yield a number of targets that could feed a validation and systems analysis pipeline.

## Immunoprecipitation-based target identification techniques

Biochemistry-based experiments have been developed to directly identify the target sequences bound by miRNAs. The first attempts of this type of assay immunoprecipitated the RISC components, and then performed microarrays or RNA sequencing to identify the captured mRNAs (Beitzinger et al., 2007; Easow et al., 2007; Zhang et al., 2007; Hendrickson et al., 2008). Such methods are promising since they should be able to identify the direct targets of mRNAs. A number of procedural modifications have improved the initial process to reduce false positive rates and increase the depth and specificity of targets discovered. These methods, referred to as Argonaute CLIP-Seq (Zisoulis et al., 2010) or Argonaute HITS-CLIP (Chi et al., 2009), utilize cross-linking prior to immunoprecipitation to firmly associate target mRNAs with miRISC. After immunoprecipitation, exposed RNA ends not covered by RISC protein are enzymatically cleaved before linkers are ligated to the bound RNA and then processed using deep sequencing. After sequencing, high tag count segments are deemed to be bonafide miRNA target sites, which are then matched computationally to individual transcripts.

Analysis of the putative recognition sites discovered by these methods indicated that not every enriched sequence has a good “seed” match to known miRNAs. This may be in part due to unknown miRNAs being present in the genome, but recent mass sequencing efforts suggest that the vast majority of miRNAs have been discovered in the major model organisms. The most likely explanation, therefore, is that the contextual cues and non-canonical pairing indeed play important roles in determining miRNA-mRNA recognition, and the data from these experiments are helping to re-define the miRNA-mRNA binding rules (Elefant et al., 2011; Wen et al., 2011).

Additional refinements to the immunoprecipitation approach have improved specificity and sensitivity. PAR-CLIP (Hafner et al., 2010) and miR-TRAP (Baigude et al., 2012) both include photoactivatable ribonucleosides in transfected miRNA mimics to allow specific cross-linking sites and higher wavelength cross-linking, which is less harmful to cells and improves RNA recovery. The PAR-CLIP method has been used to achieve single nucleotide resolution of the binding site due to the specificity of the cross-linking. Modifications to denaturing conditions and the nuclease digestion of extraneous RNA can improve data by reducing biases resulting from conditions used in previous methods (Kishore et al., 2011).

These approaches often rely on overexpression of a particular miRNA to load the RISC. The over-representation of a specific miRNA in active RISC can cause off-target interactions, possibly influenced by dosage and elevated contribution of seed sequence similarity to miRNA:mRNA association (Birmingham et al., 2006; Arvey et al., 2010). This phenomenon, however, might recapitulate the function of the overexpressed miRNA in the screen assay itself, and thus may be relevant to the identification of targets. Conversely, endogenous miRNA programmed RISC will always comprise some percentage of the total data. Both errors will introduce false positives. The miR-TRAP method seeks to avoid this issue by inclusion of a biotin tag on transfected miRNA in an effort to select only for complexes containing specific miRNAs (Baigude et al., 2012). Perhaps most promising of new technologies, crosslinking, ligation, and sequencing of hybrids (CLASH) of RNA pulled down with AGO complexes, may provide the ability to simultaneously discover mRNAs being downregulated by RISC and the specific miRNA(s) which target them, as a miRNA sequence and a fragment of its targeted RNA sequence will be ligated together and sequenced as a single chimeric sequence (Helwak et al., 2013).

Although these immunoprecipitation-based methods can provide quantitative data about miRNA-target binding, their main drawback is that they do not quantify the extent of mRNA or protein downregulation. For this reason, a combination of proteomic/transcriptomic profiing with the direct immunoprecipitation methods might offer the best quality datasets for constructing miRNA-target interaction networks. A meta analysis of microarray data from miRNA transfection experiments compared to Argonaute CLIP-Seq data not surprisingly showed only partial overlap (Wen et al., 2011), presumably reflecting the inherent biases of each method. Such discrepancies might be predictive of direct versus indirect effects of miRNAs against target mRNAs or proteins. Furthermore, investigation of the dose-dependent effects of miRNAs against targets will likely be important for appreciating how a miRNA or anti-miRNA therapeutic will behave *in vivo*, in particular whether or not there are potentially beneficial or harmful dosage effects.

## Building and validating networks

**Figure 3:**
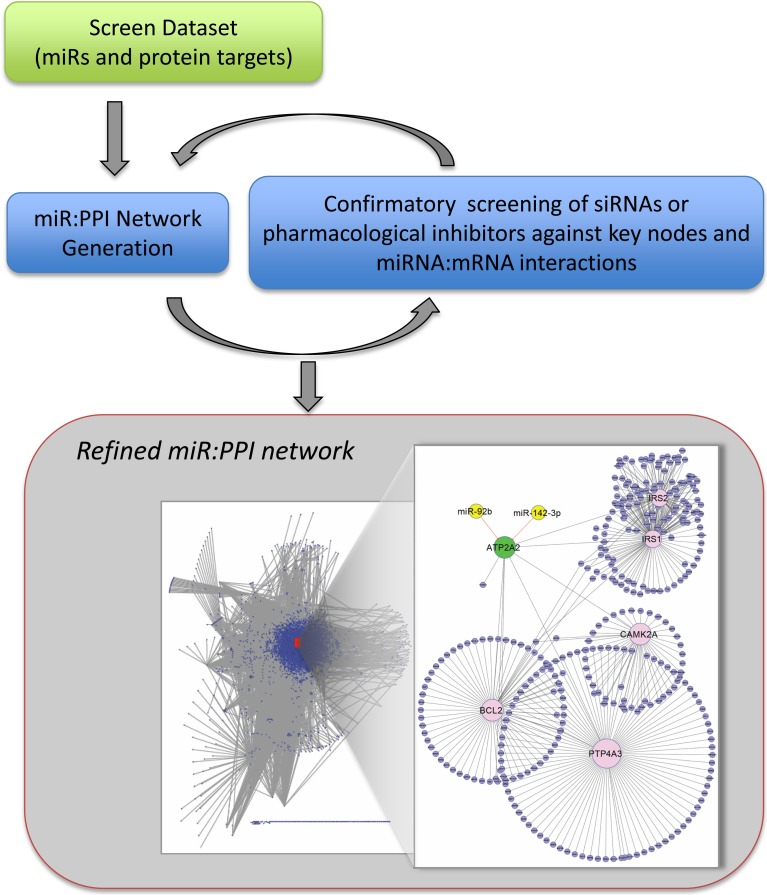
**Pipeline for iterative process of network construction and confirmatory screening of key nodes.** The screen dataset (as in Figure 1) is filtered and used for construction of the preliminary network. We propose that it is beneficial to evaluate individual protein nodes by screening specific si/shRNAs, pharmacological inhibitors or by protein overexpression. Similarly, miR:protein interactions can be validated by monitoring protein levels and direct interaction confirmed by site-directed mutagenesis of the recognition elements in the mRNAs (see text). The confirmatory cycles lead to a refined dataset and network. Statistical significance of screen hits can be relaxed because of the confirmatory process. The interactome shown contains miRNAs (yellow) found in a screen to result in SERCA2 (ATP2A2) (green) inhibition >30%, *p* < 0.05, are evolutionarily conserved, and are upregulated in human heart failure. Inset: SERCA2 (node enlarged) centric network showing interaction with miR92b and miR-142-3b that were determined by confirmatory screening to target SERCA2 (unpublished data).

Functional screening of miRNA mimics generates a list of miRNAs that, when overexpressed, affect the desired phenotype to varying degrees. In our experience, screening about 900 miRNAs in a commercial mRNA mimic collection against a phenotypic assay results in between 30 and 200 statistically significant hits, (e.g., Colas et al., 2012), consistent with results from other complex biological assays such as (Eulalio et al., 2012; Jentzsch et al., 2012). The hits can be prioritized according to experimental goals (e.g. filtered by expression within a target tissue). Once the targets are identified through the strategies described above, they can be mapped to the human PPI network. From the human PPI, a sub-network is obtained by retaining the edges in which one of the nodes is in the target list (Figure 3). This amounts to retaining all the nodes in the PPI that directly interact with at least one target gene. One can define rules about which nodes and edges from the PPI should be included. For example, one may retain only those edges in which both nodes are in the target list or those that are functionally associated. This may result in a much more sparse network.

How well do predicted networks reflect reality? A recent study Becker et al. (2012) shows that miRs are encoded in the genome as individual miRNA genes or as gene clusters and transcribed as polycistronic units. These authors estimated that about 50% of all miRNAs are co-expressed with neighboring miRNAs and, most importantly, that these clusters coordinately regulate multiple members of protein-protein interaction network clusters. Another study (Alshalalfa et al., 2012) showed that combining protein functional interaction networks with miR detection revealed several miR-regulated interaction modules that were indeed enriched in focal adhesion and prostate cancer pathways, and yet another used screen data to reveal miRNA control of p53 (Becker et al., 2012). Illustrative of such recent efforts to deduce high quality PPIs from miRNA screen datasets is the control of epithelial to mesenchymal transition by miR-200 family (Sass et al., 2011). The study first used an *in silico* approach comparing miRNA target sites from published PAR-CLIP dataset (Hafner et al., 2010) to proteomics datasets (Baek et al., 2008; Selbach et al., 2008) to conclude that miRNAs have a propensity to target proteins involved in multi-protein complexes. Furthermore, they showed that protein complexes are coordinately regulated by clusters of miRNAs, a conclusion supported by an analysis of miRNAs that regulate transcription factor response elements in cell culture (Becker et al., 2012). To probe the notion that miRNA clusters coordinately control biological processes, Sass et al. (2011) went on to show that additional members of the transcriptional complex controlling E-cadherin, in addition to previously identified members, are under coordinate control by miRNAs that reside within the miR141-200c cluster. Although these pioneering studies support the idea that combining proteomics-based target identification with a network-based strategy can be used to construct reliable miRNA:protein interaction networks, it should be emphasized that the validation has been sparse, and that large-scale approaches, such as by siRNA screening, are needed to evaluate the veracity of the regulatory networks.

## Summary and prospects

Several features of miRNAs make functional, whole miRNAome screening attractive as a platform to generate systems-level descriptions of complex biological regulatory networks and help interpret the massive transcriptome datasets emerging in all areas of biology. First, the total number of miRNAs is relatively few compared to siRNA or chemical libraries; yet, because of target recognition degeneracy, the miRNAome regulates a large proportion of the proteome. Second, since miRNA recognition of mRNA transcripts is sequence based, the identification of mRNA targets poses fewer problems than associated with identification of relevant targets of small molecules from chemical screens (Rix and Superti-Furga, 2009), although methods for high throughput identification of miRNA targets remain costly and far from robust. Third, based on co-evolution of miRNAs and the networks they control, it is tempting to speculate that the nodes targeted by the miRNAs might be selective for particular biological processes, and hence comprise good points for therapeutic intervention.

Currently, screening technology combined with the availability of miRNA and si/shRNA libraries make it straightforward to design and implement a moderate throughput whole genome miRNAome or si/shRNA transcriptome screen (Figure 1). This includes iPSC-based disease models, which offer an unprecedented ability to interrogate disease relevant processes and reveal potential new drug targets. The bottleneck today is target identification. Ideally, proteomics datasets should provide clear and consistent results from over-expression of miRNAs. Unfortunately, there is considerable variation between datasets obtained from proteomics analysis of the same miRNA assayed by overexpression in the same cells. For instance, comparison of the proteins downregulated by miR-34a (by Bargaje et al.) revealed only 5 proteins in common out of 3365 (Bargaje et al., 2012) and 1495 (Chen et al., 2011). Similarly, Shirdel et al. (Shirdel et al., 2011) compared the results of miR-124 overexpression and found only 10 common targets from 3 experiments, comprising only 3.7% of the smallest dataset. Similarly, the general conclusion about computational prediction resources is that none alone can perfectly identify mRNA targets, even when mRNAs are filtered by analysis (e.g. microarray type) and cell type (Baek et al., 2008; Selbach et al., 2008; Shirdel et al., 2011). Nonetheless, our experience is consistent with the conclusion of Shirdel et al. that the current methods are suitable to provide an initial prediction, and this is aided by recent resources such as mirGator and mirDIP that integrate several up-to-date miRNA target prediction databases. In practice, PPI networks are often constructed from targets from multiple prediction algorithms, see discussion in (Alexiou et al., 2009; Shirdel et al., 2011). Furthermore, we use moderate throughput siRNA screening against individual pathway components to confirm the validity of predicted PPIs (Figure 3) (Colas et al., 2012).

Finally, functional miRNA screening is a potentially powerful method of identifying miRNAs and PPIs that control complex biological processes. Although miRNA screening is mainly considered as a strategy to reveal miRNAs that naturally control biological processes, we propose a more expanded view, and suggest that miRNA screening also has the potential to interrogate biological networks even if the active miRNAs are not natural regulators. Like chemical and si/shRNA functional genomics screens, miRNAs screening, coupled to target identification and iterations of PPI network construction, validation and refinement, might offer an attractive pipeline to interrogate complex biology.

### Conflict of interest statement

The authors declare that the research was conducted in the absence of any commercial or financial relationships that could be construed as a potential conflict of interest.
